# *trans*-Dichloridobis(3,5-dimethyl­pyridine-κ*N*)(ethano­lato-κ*O*)oxido­rhenium(V)

**DOI:** 10.1107/S1600536811029588

**Published:** 2011-07-30

**Authors:** Anna Skarżyńska, Anna M. Trzeciak, Andrzej Gniewek

**Affiliations:** a Faculty of Chemistry, University of Wrocław, 14 F. Joliot-Curie, 50-383 Wrocław, Poland

## Abstract

The title compound, [Re(C_2_H_5_O)Cl_2_O(C_7_H_9_N)_2_], was crystallized from ethanol. The crystal structure of this complex contains a Re(V) atom in a slightly distorted octahedral coordination geometry with pairs of equivalent ligands in *trans* positions. Adjacent complex mol­ecules are linked by weak C—H⋯Cl hydrogen bonds. The crystal structure is additionally stabilized by π–π stacking inter­actions between the aromatic rings with centroid–centroid distances of 3.546 (4) Å.

## Related literature

The structure of the title compound was determined as part of a larger study on rhenium chemistry. For related structures and further discussion, see: Fortin & Beauchamp (1998 ▶); Iengo *et al.* (2001 ▶); Lock & Turner (1977 ▶). For hydrogen-bond inter­actions, see: Aullón *et al.* (1998 ▶); Bernstein *et al.* (1995 ▶); Desiraju & Steiner (1999 ▶) and for π–π stacking contacts, see: McGaughey *et al.* (1998 ▶). For details of the temperature control applied during data collection, see: Cosier & Glazer (1986 ▶) and for specifications of the analytical numeric absorption correction, see: Clark & Reid (1995 ▶).
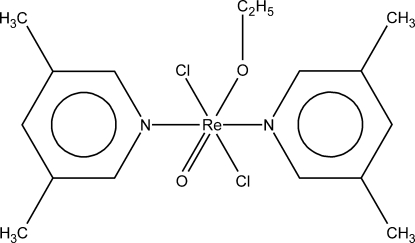


## Experimental

### 

#### Crystal data

[Re(C_2_H_5_O)Cl_2_O(C_7_H_9_N)_2_]
*M* *_r_* = 532.46
Triclinic, 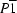

*a* = 8.782 (2) Å
*b* = 9.458 (2) Å
*c* = 12.022 (3) Å
α = 76.71 (3)°
β = 70.84 (3)°
γ = 73.21 (3)°
*V* = 892.9 (4) Å^3^
*Z* = 2
Mo *K*α radiation
μ = 7.11 mm^−1^
*T* = 90 K
0.14 × 0.10 × 0.08 mm

#### Data collection

Oxford Diffraction Xcalibur PX diffractometer with CCD detector
Absorption correction: analytical (*CrysAlis RED*; Oxford Diffraction, 2010 ▶) *T*_min_ = 0.522, *T*_max_ = 0.608
9213 measured reflections
3992 independent reflections
3519 reflections with *I* > 2σ(*I*)
*R*_int_ = 0.039

#### Refinement

*R*[*F* ^2^ > 2σ(*F* ^2^)] = 0.021
*wR*(*F* ^2^) = 0.043
*S* = 0.96
3992 reflections
213 parameters
H-atom parameters constrained
Δρ_max_ = 0.89 e Å^−3^
Δρ_min_ = −0.69 e Å^−3^

### 

Data collection: *CrysAlis CCD* (Oxford Diffraction, 2010 ▶); cell refinement: *CrysAlis RED* (Oxford Diffraction, 2010 ▶); data reduction: *CrysAlis RED*; program(s) used to solve structure: *SHELXS97* (Sheldrick, 2008 ▶); program(s) used to refine structure: *SHELXL97* (Sheldrick, 2008 ▶); molecular graphics: *ORTEP-3* (Farrugia, 1997 ▶); software used to prepare material for publication: *SHELXL97*.

## Supplementary Material

Crystal structure: contains datablock(s) global, I. DOI: 10.1107/S1600536811029588/bt5584sup1.cif

Structure factors: contains datablock(s) I. DOI: 10.1107/S1600536811029588/bt5584Isup2.hkl

Additional supplementary materials:  crystallographic information; 3D view; checkCIF report

## Figures and Tables

**Table:** 

Re—O1	1.698 (2)
Re—O2	1.882 (2)
Re—Cl1	2.4360 (11)
Re—Cl2	2.3728 (11)
Re—N1	2.143 (3)
Re—N2	2.132 (3)

**Table:** 

O1—Re—O2	171.57 (10)
N1—Re—N2	177.79 (10)
Cl1—Re—Cl2	173.74 (3)

**Table 2:** Hydrogen-bond geometry (Å, °)

*D*—H⋯*A*	*D*—H	H⋯*A*	*D*⋯*A*	*D*—H⋯*A*
C121—H12*C*⋯Cl1^i^	0.98	2.82	3.754 (4)	160
C13—H13⋯Cl1^i^	0.95	2.92	3.717 (4)	142

Symmetry code: (i) 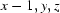.

**Fig. 1.**
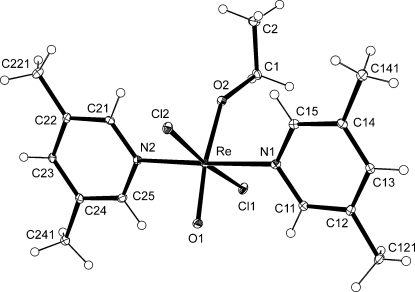
The molecular structure and atom numbering scheme of the title compound. Displacement ellipsoids are drawn at the 30% probability level and H atoms are shown as small spheres of arbitrary radii.

**Fig. 2.**
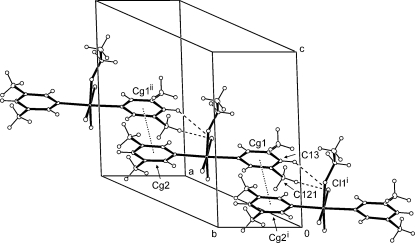
The arrangement of the molecules in the crystal structure, a perspective view in an oblique direction. Weak intermolecular hydrogen bonds are represented by dashed lines and the π-π stacking interactions by dotted lines. Cg1 and Cg2 denote the centroids of the N1/C11—C15 and N2/C21—C25 pyridine rings, respectively. For clarity, H atoms that are not involved in the discussed interactions have been omitted. [Symmetry codes: (i) x - 1, y, z; (ii) x + 1, y, z.]

## supplementary crystallographic information

### Comment

Complexes of the ReO(O*R*)*X*_2_*L*_2_ type (where *R* is an alkyl group, *X* is a halogen and *L* is an N-donor ligand or monodendate phosphine) are commonly used as precursors for other Re(V) species, such as dioxo mononuclear and dinuclear compounds (Fortin & Beauchamp, 1998). When *L* is a diaza ligand, they might also be applied for the construction of multi-metal supramolecular assemblies (Iengo *et al.*, 2001). Normally, these complexes are obtained as the all *trans* isomers (Lock & Turner, 1977). In this paper we report the synthesis procedure and crystal structure of an oxorhenium(V) complex with 3,5-dimethylpyridine ligands, the title compound.

The environment around the metal center is a slightly distorted octahedron with two chloro ligands, two 3,5-dimethylpyridine ligands, ethoxo and oxo ligands, all in *trans* positions to each other (Fig. 1). The observed Re—ligand bond distances (Table 1) are similar to the reported for analogous complexes of the ReO(O*R*)*X*_2_*L*_2_ type. However, the distortion of the angles in the coordination sphere of the Re atom is more significant than in similar compounds. Especially, the O1—Re—O2 and Cl1—Re—Cl2 angles differ from the expected value of 180°.

In the crystal structure, the molecules of the title complex are linked by a few weak hydrogen interactions of the C—H···Cl type (Desiraju & Steiner, 1999). The C13 and C121 atoms act as hydrogen-bond donors to Cl1^i^ [symmetry code: (i) *x* – 1, *y*, *z*] as an acceptor (Table 2). The observed C—H···Cl distances are similar to the values of the N—H···Cl hydrogen bonds identified for Cl bonded to a transition metal (Aullón *et al.*, 1998). Each of the molecules accepts two hydrogen bonds and also donates two hydrogen bonds (Fig. 2), thus forming a *C*(7)*C*(7)[*R*_2_^1^(6)] chain of rings motif (Bernstein *et al.*, 1995).

Additionally, the N1/C11–C15 and N2/C21–C25 rings are engaged in π-π stacking contacts (Fig. 2), which further assist in the stabilization of the crystal structure by assembling chains running parallel to the [100] direction. The distance of the centroids and the offset of the pyridine rings (Table 3) are typical for energetically favorable non-bonded aromatic interactions (McGaughey *et al.*, 1998).

### Experimental

The title compound was prepared similarly to the previously reported rhenium(V) complex with pyrazine ligands (Iengo *et al.*, 2001). 3,5-dimethylpyridine (0.55 ml, 4.77 mmol) was added to the suspension of ReOCl_3_(OPPh_3_)(SMe_2_) (1.0 g, 1.54 mmol) in absolute ethanol (10 ml). The mixture was heated under reflux for 1 h, forming dark blue solution. Upon cooling down, crystalline precipitate appeared in the system. It was filtered off, washed with ethanol and diethyl ether. Finally, the product was dried *in vacuo*. Yield: 0.70 g, 85%. Analysis calculated: C 36.09, H 4.35, N 5.26%; found: C 36.04, H 4.23, N 5.25%. IR (KBr, cm^-1^): δ(OCH_2_) 915 (*versus*), ν(Re=O) 960 (*versus*). ^1^H NMR (CDCl_3_): δ 1.00 (3*H*, t, ^3^J = 6.9 Hz, CH_3_), 2.32 (12*H*, s, CH_3_) 3.75 (2*H*, q, ^3^J = 6.9 Hz, CH_2_), 7.34 (2*H*, s, CH), 8.48 (4*H*, s, CH).

### Refinement

All H atoms were positioned geometrically and refined using a riding model with aromatic C—H = 0.95Å and *U*_iso_(H) = 1.2*U*_eq_(C). The methyl groups were refined with C—H = 0.98Å and *U*_iso_(H) = 1.5*U*_eq_(C). The highest residual peak and the deepest hole in the final difference map are located 0.97 and 1.24Å from the Re atom, respectively.

### Crystal data

**Table:**

[Re(C_2_H_5_O)Cl_2_O(C_7_H_9_N)_2_]	*Z* = 2
*M**_r_* = 532.46	*F*(000) = 516
Triclinic, *P*1	*D*_x_ = 1.980 Mg m^−^^3^
Hall symbol: -P 1	Mo *K*α radiation, λ = 0.71073 Å
*a* = 8.782 (2) Å	Cell parameters from 6128 reflections
*b* = 9.458 (2) Å	θ = 5.0–27.5°
*c* = 12.022 (3) Å	µ = 7.11 mm^−^^1^
α = 76.71 (3)°	*T* = 90 K
β = 70.84 (3)°	Block, dark blue
γ = 73.21 (3)°	0.14 × 0.10 × 0.08 mm
*V* = 892.9 (4) Å^3^	

### Data collection

**Table:**

Oxford Diffraction Xcalibur PX diffractometer with CCD detector	3992 independent reflections
Radiation source: fine-focus sealed tube	3519 reflections with *I* > 2σ(*I*)
graphite	*R*_int_ = 0.039
φ and ω scans	θ_max_ = 27.5°, θ_min_ = 5.0°
Absorption correction: analytical (*CrysAlis RED*; Oxford Diffraction, 2010)	*h* = −11→8
*T*_min_ = 0.522, *T*_max_ = 0.608	*k* = −12→12
9213 measured reflections	*l* = −15→15

### Refinement

**Table:**

Refinement on *F*^2^	Primary atom site location: heavy-atom method
Least-squares matrix: full	Secondary atom site location: difference Fourier map
*R*[*F*^2^ > 2σ(*F*^2^)] = 0.021	Hydrogen site location: inferred from neighbouring sites
*wR*(*F*^2^) = 0.043	H-atom parameters constrained
*S* = 0.96	*w* = 1/[σ^2^(*F*_o_^2^) + (0.0155*P*)^2^] where *P* = (*F*_o_^2^ + 2*F*_c_^2^)/3
3992 reflections	(Δ/σ)_max_ = 0.001
213 parameters	Δρ_max_ = 0.89 e Å^−^^3^
0 restraints	Δρ_min_ = −0.69 e Å^−^^3^

### Special details

**Table:**

Experimental. The crystal was placed in the cold stream of an open-flow nitrogen cryostat (Cosier & Glazer, 1986) operating at 90 K. Analytical numeric absorption correction was carried out with *CrysAlis RED* (Oxford Diffraction, 2010) using a multifaceted crystal model (Clark & Reid, 1995).
Geometry. All e.s.d.'s (except the e.s.d. in the dihedral angle between two l.s. planes) are estimated using the full covariance matrix. The cell e.s.d.'s are taken into account individually in the estimation of e.s.d.'s in distances, angles and torsion angles; correlations between e.s.d.'s in cell parameters are only used when they are defined by crystal symmetry. An approximate (isotropic) treatment of cell e.s.d.'s is used for estimating e.s.d.'s involving l.s. planes.
Refinement. Refinement of *F*^2^ against ALL reflections. The weighted *R*-factor *wR* and goodness of fit *S* are based on *F*^2^, conventional *R*-factors *R* are based on *F*, with *F* set to zero for negative *F*^2^. The threshold expression of *F*^2^ > 2σ(*F*^2^) is used only for calculating *R*-factors(gt) *etc*. and is not relevant to the choice of reflections for refinement. *R*-factors based on *F*^2^ are statistically about twice as large as those based on *F* and *R*- factors based on ALL data will be even larger.

### Fractional atomic coordinates and isotropic or equivalent isotropic displacement parameters (Å^2^)

**Table:**

	*x*	*y*	*z*	*U*_iso_*/*U*_eq_	
Re	0.631394 (17)	0.243503 (16)	0.216648 (12)	0.00894 (4)	
Cl1	0.77034 (10)	−0.01144 (9)	0.27897 (7)	0.01403 (17)	
Cl2	0.48876 (10)	0.49592 (9)	0.17785 (7)	0.01358 (17)	
O1	0.6619 (3)	0.1964 (3)	0.0816 (2)	0.0130 (5)	
O2	0.5921 (3)	0.2674 (3)	0.37560 (19)	0.0115 (5)	
N1	0.4009 (3)	0.1765 (3)	0.2774 (2)	0.0104 (6)	
N2	0.8601 (3)	0.3103 (3)	0.1631 (2)	0.0101 (6)	
C1	0.5564 (4)	0.1947 (4)	0.4951 (3)	0.0136 (7)	
H1A	0.6603	0.1316	0.5120	0.016*	
H1B	0.4811	0.1290	0.5067	0.016*	
C2	0.4763 (5)	0.3073 (4)	0.5805 (3)	0.0188 (8)	
H2A	0.5537	0.3681	0.5727	0.028*	
H2B	0.4476	0.2549	0.6623	0.028*	
H2C	0.3756	0.3721	0.5621	0.028*	
C11	0.3875 (4)	0.0612 (4)	0.2362 (3)	0.0106 (7)	
H11	0.4825	0.0094	0.1829	0.013*	
C12	0.2406 (4)	0.0147 (4)	0.2683 (3)	0.0112 (7)	
C13	0.1050 (4)	0.0892 (4)	0.3481 (3)	0.0114 (7)	
H13	0.0028	0.0598	0.3719	0.014*	
C14	0.1167 (4)	0.2065 (4)	0.3940 (3)	0.0121 (7)	
C15	0.2686 (4)	0.2471 (4)	0.3544 (3)	0.0107 (7)	
H15	0.2783	0.3285	0.3834	0.013*	
C121	0.2334 (4)	−0.1124 (4)	0.2158 (3)	0.0150 (7)	
H12A	0.2820	−0.2074	0.2582	0.022*	
H12B	0.2960	−0.1038	0.1314	0.022*	
H12C	0.1178	−0.1087	0.2237	0.022*	
C141	−0.0273 (4)	0.2905 (4)	0.4818 (3)	0.0182 (8)	
H14A	−0.1294	0.2644	0.4860	0.027*	
H14B	−0.0380	0.3981	0.4559	0.027*	
H14C	−0.0080	0.2636	0.5606	0.027*	
C21	0.8737 (4)	0.4278 (4)	0.2016 (3)	0.0103 (7)	
H21	0.7792	0.4800	0.2549	0.012*	
C22	1.0199 (4)	0.4760 (4)	0.1666 (3)	0.0102 (7)	
C23	1.1549 (4)	0.4001 (4)	0.0868 (3)	0.0107 (7)	
H23	1.2563	0.4313	0.0603	0.013*	
C24	1.1439 (4)	0.2788 (4)	0.0452 (3)	0.0107 (7)	
C25	0.9937 (4)	0.2371 (4)	0.0874 (3)	0.0126 (7)	
H25	0.9848	0.1529	0.0615	0.015*	
C221	1.0287 (4)	0.6068 (4)	0.2129 (3)	0.0157 (7)	
H22A	1.1026	0.5727	0.2645	0.024*	
H22B	0.9176	0.6537	0.2586	0.024*	
H22C	1.0716	0.6796	0.1459	0.024*	
C241	1.2877 (4)	0.1938 (4)	−0.0404 (3)	0.0147 (7)	
H24A	1.2542	0.1140	−0.0594	0.022*	
H24B	1.3792	0.1501	−0.0042	0.022*	
H24C	1.3239	0.2616	−0.1135	0.022*	

### Atomic displacement parameters (Å^2^)

**Table:**

	*U*^11^	*U*^22^	*U*^33^	*U*^12^	*U*^13^	*U*^23^
Re	0.00818 (7)	0.00968 (7)	0.00966 (7)	−0.00304 (5)	−0.00212 (5)	−0.00210 (5)
Cl1	0.0115 (4)	0.0100 (4)	0.0204 (4)	−0.0022 (3)	−0.0049 (3)	−0.0020 (3)
Cl2	0.0124 (4)	0.0107 (4)	0.0170 (4)	−0.0025 (3)	−0.0045 (3)	−0.0006 (3)
O1	0.0115 (12)	0.0156 (12)	0.0131 (12)	−0.0047 (10)	−0.0018 (10)	−0.0049 (10)
O2	0.0126 (12)	0.0117 (11)	0.0110 (11)	−0.0035 (10)	−0.0044 (10)	−0.0007 (10)
N1	0.0112 (15)	0.0110 (14)	0.0105 (13)	−0.0018 (12)	−0.0063 (12)	−0.0006 (11)
N2	0.0107 (14)	0.0111 (14)	0.0092 (13)	−0.0032 (12)	−0.0037 (11)	−0.0010 (11)
C1	0.0151 (18)	0.0121 (17)	0.0105 (16)	−0.0037 (15)	−0.0010 (14)	0.0012 (14)
C2	0.025 (2)	0.0162 (18)	0.0134 (17)	−0.0051 (16)	−0.0011 (16)	−0.0042 (15)
C11	0.0105 (17)	0.0099 (16)	0.0103 (16)	−0.0013 (14)	−0.0032 (13)	−0.0005 (13)
C12	0.0132 (18)	0.0108 (16)	0.0118 (16)	−0.0045 (14)	−0.0068 (14)	0.0011 (14)
C13	0.0102 (17)	0.0127 (16)	0.0102 (16)	−0.0035 (14)	−0.0015 (14)	−0.0009 (14)
C14	0.0098 (17)	0.0140 (17)	0.0112 (16)	−0.0013 (14)	−0.0022 (14)	−0.0025 (14)
C15	0.0114 (17)	0.0095 (16)	0.0110 (16)	0.0003 (14)	−0.0055 (14)	−0.0010 (13)
C121	0.0136 (18)	0.0126 (17)	0.0197 (18)	−0.0048 (15)	−0.0018 (15)	−0.0063 (15)
C141	0.0164 (19)	0.0175 (18)	0.0202 (19)	−0.0063 (16)	0.0004 (15)	−0.0069 (16)
C21	0.0108 (17)	0.0114 (16)	0.0079 (15)	−0.0013 (14)	−0.0031 (13)	−0.0009 (13)
C22	0.0136 (17)	0.0088 (16)	0.0098 (15)	−0.0038 (14)	−0.0068 (14)	0.0027 (13)
C23	0.0075 (17)	0.0117 (16)	0.0126 (16)	−0.0036 (14)	−0.0020 (14)	−0.0008 (14)
C24	0.0139 (18)	0.0105 (16)	0.0076 (15)	−0.0037 (14)	−0.0053 (14)	0.0031 (13)
C25	0.0160 (18)	0.0117 (16)	0.0118 (16)	−0.0042 (15)	−0.0051 (14)	−0.0022 (14)
C221	0.0169 (19)	0.0142 (17)	0.0196 (18)	−0.0047 (15)	−0.0075 (15)	−0.0047 (15)
C241	0.0142 (18)	0.0167 (18)	0.0133 (17)	−0.0065 (15)	−0.0004 (14)	−0.0042 (15)

### Geometric parameters (Å, °)

**Table:**

Re—O1	1.698 (2)	C14—C141	1.508 (5)
Re—O2	1.882 (2)	C15—H15	0.9500
Re—Cl1	2.4360 (11)	C121—H12A	0.9800
Re—Cl2	2.3728 (11)	C121—H12B	0.9800
Re—N1	2.143 (3)	C121—H12C	0.9800
Re—N2	2.132 (3)	C141—H14A	0.9800
O2—C1	1.418 (4)	C141—H14B	0.9800
N1—C15	1.334 (4)	C141—H14C	0.9800
N1—C11	1.345 (4)	C21—C22	1.389 (4)
N2—C25	1.343 (4)	C21—H21	0.9500
N2—C21	1.346 (4)	C22—C23	1.385 (4)
C1—C2	1.510 (5)	C22—C221	1.500 (5)
C1—H1A	0.9900	C23—C24	1.392 (4)
C1—H1B	0.9900	C23—H23	0.9500
C2—H2A	0.9800	C24—C25	1.387 (4)
C2—H2B	0.9800	C24—C241	1.497 (5)
C2—H2C	0.9800	C25—H25	0.9500
C11—C12	1.390 (4)	C221—H22A	0.9800
C11—H11	0.9500	C221—H22B	0.9800
C12—C13	1.383 (4)	C221—H22C	0.9800
C12—C121	1.504 (5)	C241—H24A	0.9800
C13—C14	1.389 (4)	C241—H24B	0.9800
C13—H13	0.9500	C241—H24C	0.9800
C14—C15	1.397 (4)		
			
O1—Re—N1	88.90 (10)	C13—C14—C141	122.6 (3)
O2—Re—N1	85.89 (10)	C15—C14—C141	120.0 (3)
O1—Re—N2	93.24 (10)	N1—C15—C14	122.8 (3)
O2—Re—N2	91.92 (10)	N1—C15—H15	118.6
O1—Re—O2	171.57 (10)	C14—C15—H15	118.6
N1—Re—N2	177.79 (10)	C12—C121—H12A	109.5
O1—Re—Cl1	88.90 (8)	C12—C121—H12B	109.5
O2—Re—Cl1	84.42 (8)	H12A—C121—H12B	109.5
N1—Re—Cl1	89.20 (8)	C12—C121—H12C	109.5
N2—Re—Cl1	90.33 (8)	H12A—C121—H12C	109.5
O1—Re—Cl2	97.31 (8)	H12B—C121—H12C	109.5
O2—Re—Cl2	89.33 (8)	C14—C141—H14A	109.5
N1—Re—Cl2	90.07 (8)	C14—C141—H14B	109.5
N2—Re—Cl2	90.16 (8)	H14A—C141—H14B	109.5
Cl1—Re—Cl2	173.74 (3)	C14—C141—H14C	109.5
C1—O2—Re	144.0 (2)	H14A—C141—H14C	109.5
C15—N1—C11	118.7 (3)	H14B—C141—H14C	109.5
C15—N1—Re	121.9 (2)	N2—C21—C22	122.7 (3)
C11—N1—Re	119.4 (2)	N2—C21—H21	118.6
C25—N2—C21	118.5 (3)	C22—C21—H21	118.6
C25—N2—Re	120.2 (2)	C23—C22—C21	117.6 (3)
C21—N2—Re	121.3 (2)	C23—C22—C221	121.8 (3)
O2—C1—C2	110.8 (3)	C21—C22—C221	120.6 (3)
O2—C1—H1A	109.5	C22—C23—C24	120.8 (3)
C2—C1—H1A	109.5	C22—C23—H23	119.6
O2—C1—H1B	109.5	C24—C23—H23	119.6
C2—C1—H1B	109.5	C25—C24—C23	117.3 (3)
H1A—C1—H1B	108.1	C25—C24—C241	120.5 (3)
C1—C2—H2A	109.5	C23—C24—C241	122.2 (3)
C1—C2—H2B	109.5	N2—C25—C24	123.0 (3)
H2A—C2—H2B	109.5	N2—C25—H25	118.5
C1—C2—H2C	109.5	C24—C25—H25	118.5
H2A—C2—H2C	109.5	C22—C221—H22A	109.5
H2B—C2—H2C	109.5	C22—C221—H22B	109.5
N1—C11—C12	122.7 (3)	H22A—C221—H22B	109.5
N1—C11—H11	118.7	C22—C221—H22C	109.5
C12—C11—H11	118.7	H22A—C221—H22C	109.5
C13—C12—C11	117.7 (3)	H22B—C221—H22C	109.5
C13—C12—C121	122.5 (3)	C24—C241—H24A	109.5
C11—C12—C121	119.8 (3)	C24—C241—H24B	109.5
C12—C13—C14	120.6 (3)	H24A—C241—H24B	109.5
C12—C13—H13	119.7	C24—C241—H24C	109.5
C14—C13—H13	119.7	H24A—C241—H24C	109.5
C13—C14—C15	117.4 (3)	H24B—C241—H24C	109.5
			
N2—Re—O2—C1	−125.9 (4)	N1—C11—C12—C13	−1.6 (5)
N1—Re—O2—C1	53.9 (4)	N1—C11—C12—C121	178.2 (3)
Cl2—Re—O2—C1	144.0 (3)	C11—C12—C13—C14	0.1 (5)
Cl1—Re—O2—C1	−35.7 (3)	C121—C12—C13—C14	−179.7 (3)
O1—Re—N1—C15	−145.0 (2)	C12—C13—C14—C15	1.3 (5)
O2—Re—N1—C15	41.7 (2)	C12—C13—C14—C141	−179.6 (3)
Cl2—Re—N1—C15	−47.7 (2)	C11—N1—C15—C14	0.0 (4)
Cl1—Re—N1—C15	126.1 (2)	Re—N1—C15—C14	179.2 (2)
O1—Re—N1—C11	34.2 (2)	C13—C14—C15—N1	−1.3 (5)
O2—Re—N1—C11	−139.2 (2)	C141—C14—C15—N1	179.5 (3)
Cl2—Re—N1—C11	131.5 (2)	C25—N2—C21—C22	−0.2 (4)
Cl1—Re—N1—C11	−54.7 (2)	Re—N2—C21—C22	−178.7 (2)
O1—Re—N2—C25	−35.3 (2)	N2—C21—C22—C23	1.3 (4)
O2—Re—N2—C25	138.1 (2)	N2—C21—C22—C221	−179.5 (3)
Cl2—Re—N2—C25	−132.6 (2)	C21—C22—C23—C24	−0.9 (5)
Cl1—Re—N2—C25	53.7 (2)	C221—C22—C23—C24	179.9 (3)
O1—Re—N2—C21	143.2 (2)	C22—C23—C24—C25	−0.5 (5)
O2—Re—N2—C21	−43.4 (2)	C22—C23—C24—C241	−179.6 (3)
Cl2—Re—N2—C21	45.9 (2)	C21—N2—C25—C24	−1.3 (4)
Cl1—Re—N2—C21	−127.9 (2)	Re—N2—C25—C24	177.2 (2)
Re—O2—C1—C2	−157.5 (3)	C23—C24—C25—N2	1.6 (5)
C15—N1—C11—C12	1.5 (4)	C241—C24—C25—N2	−179.2 (3)
Re—N1—C11—C12	−177.7 (2)		

### Hydrogen-bond geometry (Å, °)

**Table:**

*D*—H···*A*	*D*—H	H···*A*	*D*···*A*	*D*—H···*A*
C121—H12C···Cl1^i^	0.98	2.82	3.754 (4)	160
C13—H13···Cl1^i^	0.95	2.92	3.717 (4)	142

Symmetry codes: (i) *x*−1, *y*, *z*.

### Table 3 Intermolecular π-π interactions (Å, °).

**Table:**

CgI	CgJ	Cg···Cg	Dihedral angle	Interplanar distance	Offset
1	2^i^	3.546 (4)	2.0 (3)	3.381 (4)	1.069 (4)
2	1^ii^	3.546 (4)	2.0 (3)	3.362 (4)	1.127 (4)

Cg1 denotes the centroid of ring N1/C11-C15; Cg2 of ring N2/C21-C25. Cg···Cg is the distance between ring centroids. The dihedral plane is that between the CgI and CgJ planes. The interplanar distance is the perpendicular distance of CgI from ring J plane. The offset is the lateral displacement of ring I relative to ring J.Symmetry codes: (i) x-1, y, z; (ii) x+1, y, z.
